# A cross-sectional survey of knowledge and attitudes towards scabies control in Australian aged care facilities

**DOI:** 10.1017/S0950268824001377

**Published:** 2024-10-21

**Authors:** Isabelle Lightbody, Skye Cash-Deans, Cielo Pasay, Florin Oprescu, Kate Mounsey

**Affiliations:** 1 School of Health, University of the Sunshine Coast, Maroochydore, QLD, Australia; 2 Infectious Diseases, QIMR Berghofer Medical Research Institute, Brisbane, QLD, Australia; 3 Sunshine Coast Health Institute, Birtinya, QLD, Australia

**Keywords:** aged care, education, infection control, KAP, nursing homes, *Sarcoptes scabiei*, scabies

## Abstract

Scabies outbreaks cause significant morbidity and disruption in aged care facilities and other institutional settings. Failure to manage scabies outbreaks may be attributable to low awareness amongst healthcare workers. A survey was distributed to healthcare workers across aged care facilities in South-East Queensland, Australia. The survey captured demographics, prior scabies experience, knowledge-based questions, and attitudes. Scabies was common in aged care facilities, with 41% of 128 respondents encountering the disease while working in aged care. Participants demonstrated sound theoretical knowledge regarding scabies (median knowledge score 82%). Scabies knowledge was not associated with years of experience in the sector or educational level but was associated with respondent age (*p* = 0.017). Knowledge gaps were evident regarding diagnosis, incubation periods, and treatment. Respondents demonstrated an inconsistent ability to identify atypical clinical presentations of scabies, showing discordance between theoretical knowledge and its practical application. The ability to identify crusted scabies was low, reflecting the high frequency of misdiagnosis of index cases in scabies outbreaks. Respondents considered scabies to be a problem and were supportive of improved management guidelines. These study outcomes will inform the design of accessible, targeted educational resources for scabies to help prevent and reduce the impact of outbreaks.

## Background

Scabies is caused by the ectoparasitic mite *Sarcoptes scabiei*, which lives and reproduces in the epidermis. Scabies has a low mortality risk but considerable morbidity, particularly when associated with further secondary infection [1]. Clinical manifestations, which generally develop 4–6 weeks after primary exposure, include pruritis and characteristic skin lesions such as nodules and vesicles [2]. Infection is most common in cavities such as between the fingers, groin areas, armpits, and genitals, as well as the wrists and breasts [3].

Though most clinical presentations are classified as ‘ordinary’ or ‘classical’ scabies, which is associated with relatively low mite burden, individuals may develop crusted scabies, characterized by hyperproliferation of the scabies mite. Crusted scabies varies in appearance but may present with deep skin fissures and hyperkeratosis, commonly on the hands and feet [3, 4]. A single infected individual may host thousands of mites, and thus index cases of crusted scabies are commonly associated with scabies outbreaks in institutional settings.

Key epidemiological studies have been undertaken to explore the disproportionately high prevalence of scabies outbreaks in aged-care facilities, where attack rates can be as high as 50% [5]. A retrospective review identified misdiagnosis of scabies as a contributing factor to 43% of outbreaks, with 83% of the index cases presenting with crusted scabies [6]. Compared to ordinary scabies, crusted scabies lesions can occur in atypical locations such as the scalp and toenails. Delayed detection and suboptimal management of scabies outbreaks may be attributable to crusted and/or atypical presentations of scabies, including in areas usually covered by clothing [3]. Delayed diagnosis may also stem from the uncertainty of residential care workers in identifying scabies, who refer their residents to doctors for diagnosis before commencing mediative strategies [6, 7]. Finally, scabies outbreaks in aged care facilities are exacerbated by both the physical and social vulnerability of the residents [5]. Residents are more likely to be exposed to the disease, more likely to acquire it, and less able to voice their distress. Residents with co-morbidities such as dementia pose a particular risk of chronic scabies infection and challenges with treatment [8].

In this work, we explore the knowledge, attitudes, and experiences of aged-care workers towards scabies. The overall aim of this research is to improve prevention and management strategies for scabies in aged care facilities by developing informed and targeted educational resources.

## Methods

### Ethics

This study was approved by the Human Research Ethics Committees of the University of the Sunshine Coast (A13513) and Uniting Care Queensland (15413).

### Survey design and deployment

Data was collected via an anonymous, 28-question cross-sectional survey during 2013 and 2014. Surveys were paper-based and took approximately 10–15 min to complete. Questions were developed based on an initial literature review analysing institutional scabies outbreaks from 1984 to 2013 [6]. Questions were reviewed for clarity and readability by content experts, students, and laypeople and refined after feedback. The survey was divided into four sections. Section 1 included demographics, role in aged care, experience in sector, education level, and experiences with scabies. Section 2 included multiple choice and true/false questions on scabies knowledge, including questions relating to scabies transmission, diagnosis, control, and treatment (Supplementary Table S1). Section 3 included numeric rating scale questions regarding personal attitudes towards scabies, as well as two questions regarding preferences for resources. A section for extra comments and feedback was also included. In Section 4, participants were asked to identify scabies cases from a series of images. Two representative images of skin with crusted scabies and three images of skin with ordinary scabies were included. Three additional images depicted impetigo, eczema, and dry skin (Supplementary Material S1). Participants were asked to tick images that they believed to be scabies cases.

Aged-care facilities in the Sunshine Coast region of Southeast Queensland, Australia, were invited to participate via phone and/or email to management. Eight high-care facilities agreed to participate, and a total of 680 surveys were deployed. Surveys were distributed with the recommendation to leave in a staff-only communal area (such as a break room) for staff to complete at their leisure. Surveys were anonymous and could not be linked back to individuals or facilities. Reply-paid envelopes were included for staff to individually mail back surveys to the investigators. One centre elected to collect in a central box and return in batches.

### Analysis

Survey data was collated and analysed using SPSS v.28 (IBM). For multiple choice and Likert scale questions, no-response answers were coded as missing values. Participants who did not tick any images in Section 4 were coded as missing values. Missing values were excluded from all percentage and frequency calculations.

#### Knowledge analysis

A knowledge construct score was developed from 11 knowledge-based multiple-choice questions (Table 2, Supplementary Material S1). The construct score was equal to the number of correct answers selected by each participant. A missing value for any of the 11 construct-score questions resulted in a construct score not being calculated for that participant. The Kruskal–Wallis test was used to determine whether the distribution of construct scores varied within levels of different demographic variables, such as age, role in aged care, time worked in the sector, and education status. The Mann–Whitney *U* test was used to determine whether the construct score distributions varied within binomial explanatory variables, such as whether the respondent had prior exposure to or experience with scabies. Each individual component of the construct score was then run using the same analyses to determine whether any individual questions, such as those relating to treatment or identification, were influenced by demographic factors.

Respondents were presented with eight images showing dermatological conditions, including examples of crusted and ordinary scabies, and asked to select which images depicted scabies. Responses were coded as selected (1), not selected (0), or missing value (999) when an unclear selection had been made. Respondents who did not identify any of the images were allocated missing values for all images (i.e. were deemed as not participating in this section of the survey). The proportion of participants who identified each image correctly and incorrectly was determined. Where this section of the survey was completed, the differences in proportions of correct answers between images were evaluated using Cochran’s *Q* test. Post hoc analyses were evaluated using Dunn’s test. McNemar’s test was used to determine if there was a statistically significant difference in the proportion of people that believed scabies could affect the scalp and the proportion that could correctly identify a scabies presentation of the scalp when provided with an image.

#### Attitudes analysis

Respondents were provided with numeric rating scale questions assessing how problematic the respondents believed scabies outbreaks were and how comfortable they would feel managing them. For each question, Wilcoxon’s sign rank test was used to compare participant’s median responses to a hypothesized neutral (3) response. For each question, the Mann–Whitney *U* test was used to compare the distribution of answers between binomial variables relating to scabies exposure. These included whether the respondent had experienced ordinary scabies, crusted scabies, or a scabies outbreak. This was used to determine whether a participant’s prior experiences with scabies influenced their perceptions of it.

## Results

### Demographics

Of the 680 surveys deployed, 128 responses were received, for a response rate of 18.8% (Table 1). Most respondents were over 40 years of age (72.3%), were female (84.2%), and were either patient care assistants (56.3%) or registered or enrolled nurses (15.9%). Most (71.9%) reported frequently working with patients requiring physical assistance. The level of experience within the aged-care sector was evenly distributed, and 62.9% of respondents had obtained a diploma/certificate qualification or higher.

**Table 1.** Participants’ characteristics

	Frequency	Per cent of respondents^a^
Overall responses received	128	
Age group		
18–24 years	15	13.4%
25–39 years	16	14.3%
40+ years	81	72.3%
No response	16	
Gender		
Female	64	84.2%
Male	12	15.8%
No response	52	
Highest educational level		
Did not complete high school	12	9.7%
Completed high school	34	27.4%
Diploma/certificate	55	44.4%
Bachelor’s degree university qualification	17	13.7%
Post–graduate university qualification	6	4.8%
No response	4	
Occupational role		
Patient care assistant	71	56.3%
Enrolled or registered nurse	20	15.9%
Allied health practitioner	1	0.8%
Support services	7	5.6%
Management/administration	9	7.1%
Other (please specify)	18	14.3%
No response	2	
Interaction with patients requiring physical assistance		
Never	11	8.6%
Rarely	6	4.7%
Sometimes	19	14.8%
Often	36	28.1%
Always	56	43.8%
No response	0	
Experience in sector		
<1 year	27	21.1%
1–4 years	34	26.6%
5–9 years	28	21.9%
10–19 years	22	17.2%
20+ years	17	13.3%
No response	0	

a Missing (‘no response’) values were excluded from percentage and frequency calculations.

### Prior experience and observations of scabies

**Figure 1.**
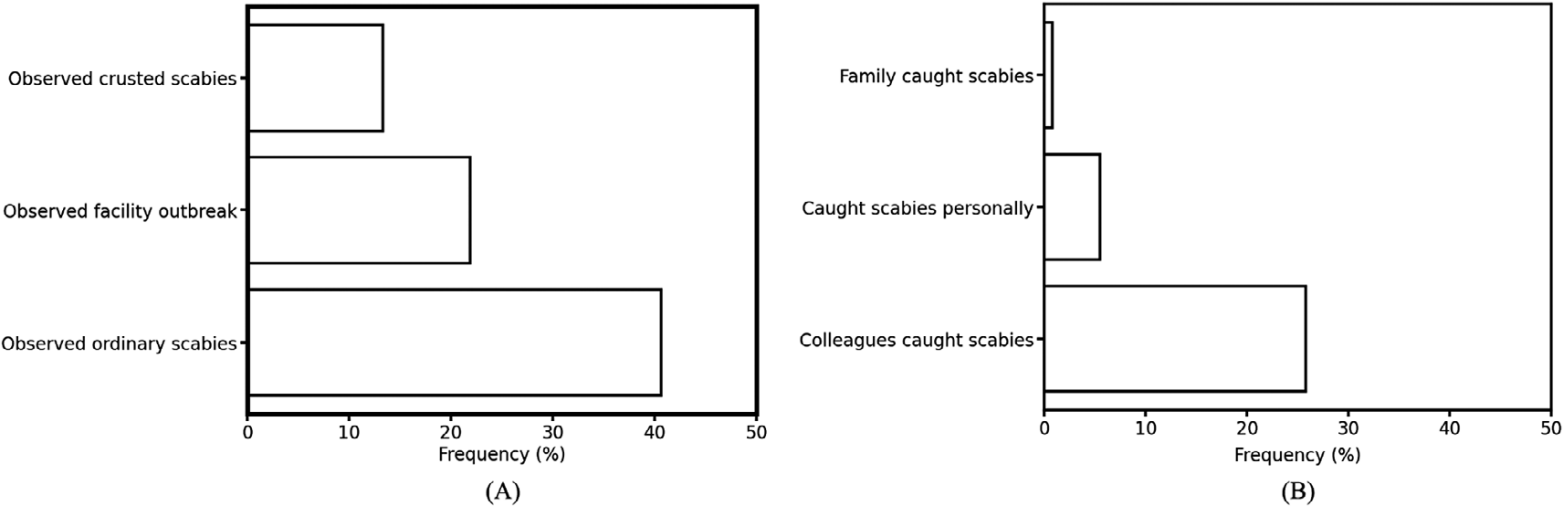
Survey participants prior experiences of scabies. The proportion of respondents that had observed scabies in their workplace during their time working in aged care (a) or reported or observed occupational exposure to scabies (b). This includes those who said their family members had caught scabies from them via their occupation. *n* = 128, missing responses were treated as a ‘no’ response.

Episodes of scabies were common in aged care facilities. Respondents had asked whether they had ever encountered scabies while working in aged care, with 41% encountering individuals with ordinary scabies, 13% with crusted scabies, and 22% scabies outbreaks on one or more occasions (Figure 1a). Of those answering yes to any of the above (*n* = 99), (32.3%) had observed scabies within the 2 years prior to the survey, while 21.2% observed it >2 years but within the last 10 years. 8.1% had observed scabies within the 6 months prior to the survey, and 38.4% of those who had observed scabies did not provide time-specific information. Reports of occupational transmission were lower, with only 6% reporting to have acquired scabies through their workplace, but a higher number (25%) reported knowing colleagues that had caught scabies through their occupation (Figure 1b).

### Knowledge

#### Overall scabies construct score

**Figure 2.**
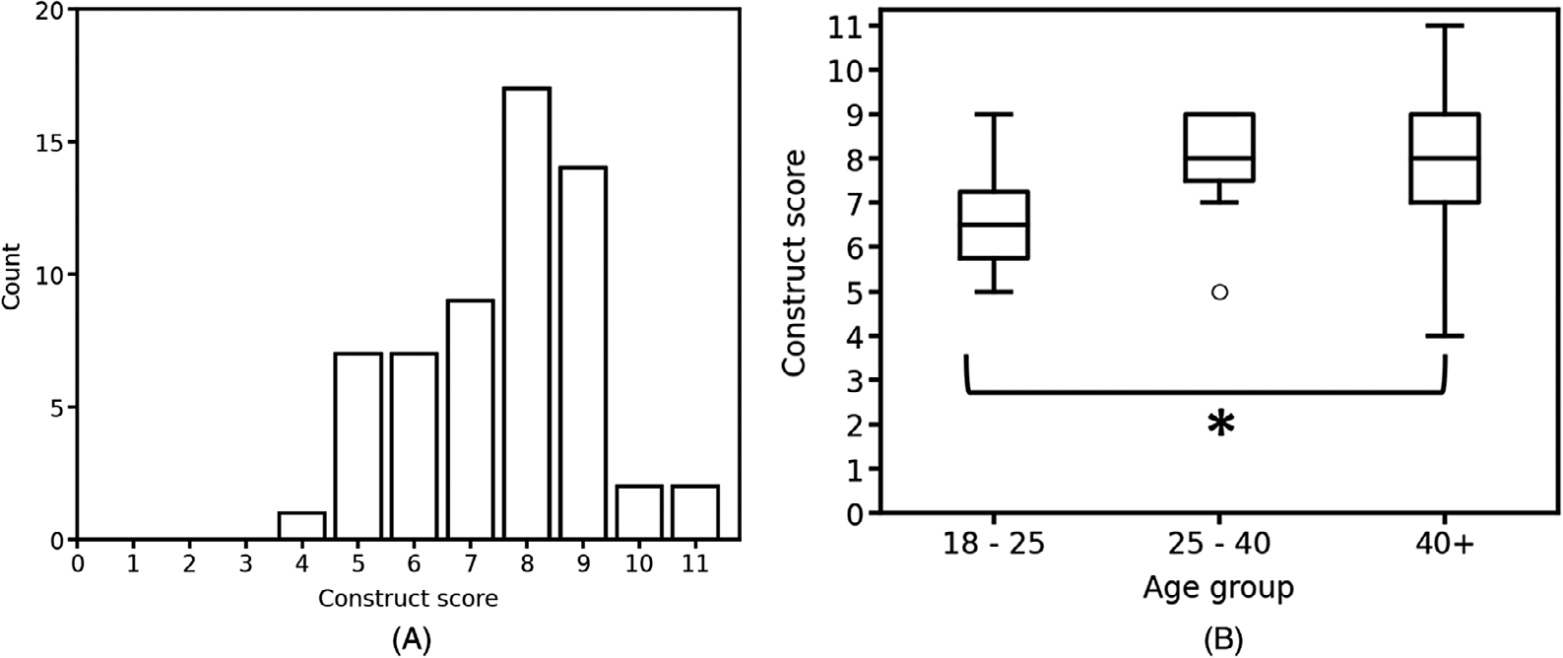
Survey participants’ knowledge of scabies. Distributions of overall knowledge construct scores (a) and across age groups (b). *p = 0.017, *n* = 59.

Construct scores were calculated for participants where all knowledge questions were completed (*n* = 59). Respondents had a median construct score of 9 out of a possible 11, or 82% (Figure 2). The Kruskal–Wallis test found no statistically significant difference between construct scores and different roles in aged care (*K* = 3.086, *n* = 57, *p* = 0.544), length of time worked in aged care (*K* = 6.821, *n* = 59, *p* = 0.146) or education status (*K* = 1.631, *n* = 59, *p* = 0.803). However, there was a small but statistically significant difference found in the construct score distributions of different ages of the respondents (*K* = 8.142, *n* = 59, *p* = 0.017). Dunn’s post-hoc analysis revealed that the 40+ age group demonstrated statistically higher levels of knowledge than the 18–25 age group (*p* = 0.05, Figure 2b). Separate analysis of the 40+ age group confirmed there was no significant difference between the median construct score and the number of years worked in aged care within the (*K* = 0.317, *n* = 39, *p* = 0 0.989). The Mann–Whitney *U* test found that prior experience with scabies had no impact on the distribution of the construct scores, whether that be from participants having caught scabies themselves (*U* = 35.5, *n* = 59, *p* = 0.357), having colleagues that had caught scabies (*U* = 269.0, *n* = 57, *p* = 0.544), or having family members that had caught scabies (*U* = 26.0, *n* = 58, *p* = 0.879). For individual questions comprising the construct score (Table 2), participants showed high awareness of topics such as scabies transmission and prevention of transmission (64.8% and 85.2% respectively), decontamination practices (83.6%) and symptoms (91.4%). Uncertainty was evident around the most appropriate and relevant diagnostic techniques (18.5% correct) and incubation periods (44.5% correct). A relatively high proportion (34.4%) of respondents incorrectly thought that poor hygiene caused scabies.

**Table 2.** Results of knowledge questionnaire used in calculation of knowledge construct score and clinical identification. Full questions and correct answers are in Supplementary Material S1

Question	Total responses (*n*)	% correct^a^ (proportion from total 128 participants)	% correct^b^ (proportion of total responses)
Scabies knowledge questions:			
A. Transmission			
Scabies is most commonly transmitted by:	88	64.8	94.3
Poor hygiene causes people to develop scabies (T/F)	109	50.8	59.6
The following factors are important to prevent scabies transmission	121	85.2	90.1
B. Clinical presentation and diagnosis			
Itching is the most common symptom of scabies	125	91.4	93.6
If mites are not found in skin scrapings, then the patient does not have scabies (T/F)	119	72.7	78.2
Scabies is most likely to be detected by	105	18.8	22.9
Scabies never affects the scalp or face (T/F)	123	75.8	78.9
Clinically, scabies presents in the following ways?	115	68	75.7
Scabies symptoms appear quickly after contact with an infected person (T/F)	124	44.5	46
C. Management and treatment			
The following are effective treatments for scabies:	115	62.5	69.6
Bedding from a scabies patient is best treated by:	119	83.6	89.9
D. Clinical identification^c^			
Impetigo	99	64.8	83.8
Dry skin	99	71.9	92.9
Infected eczema	99	42.2	54
Ordinary scabies, elderly skin, scratch marks	97	41.4	54.6
OS, axilla	99	52.3	67.7
OS, wrists, burrows visible	99	69.5	89.9
CS, infected, toes	99	13.3	17.2
CS, scalp	98	24.2	31.6

a For these proportions, non-completion of a question was assigned as an incorrect response.

b As not all participants answered all questions, this proportion was calculated on the numbers of participants that responded to this question.

c This section was only attempted by 99 participants. Where the *n* total responses is less than 99, the response was ambiguous (such as a question mark instead of a tick/circle).

#### Clinical identification of scabies

**Figure 3.**
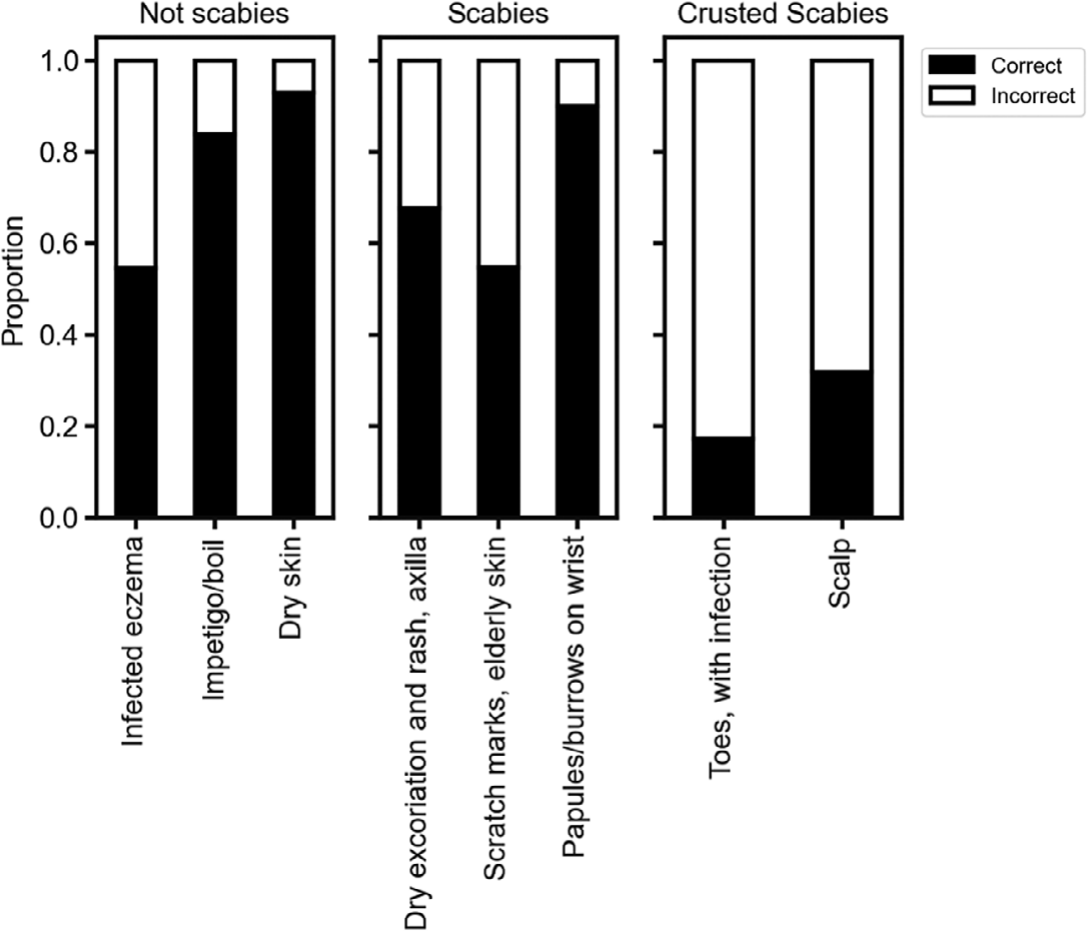
Ability of survey participants to correctly identify representative images of scabies. Respondents were shown a series of colour images representing non-scabies (‘not scabies’ panel), ordinary scabies (‘scabies’ panel) and crusted scabies (‘crusted scabies’ panel) and asked to distinguish which images were suggestive of scabies. The *x*-axis shows a description of the images, and *y*-axis proportions of correct versus incorrect. Only respondents making an attempt at this section of the survey were included in calculation of correct/incorrect proportions (*n* = 99).

In this section of the survey, respondents were asked to identify which clinical images shown depicted scabies. Only 99 of the 128 respondents completed this part of the survey (i.e. for values classed as ‘missing’, no part of the survey page was marked). The results of Cochran’s *Q* test showed that there was a statistically significant difference in the proportions of correctly identified conditions (*Q* = 226.132, *n* = 99, *p* < 0.001) (Figure 3). This meant that respondents were able to identify some conditions as being scabies/not scabies better than others. Post hoc analysis determined no statistically significant differences in the proportions of correctly identified conditions between images depicting dry skin vs. crusted scabies of toes (*p* = 0.078), dry skin vs. impetigo/boil (*p* = 0.240), crusted scabies of toes vs. impetigo/boil (*p* = 0.769), ordinary scabies on elderly skin vs. ordinary scabies of axilla with rash (*p* = 0.078), ordinary scabies on elderly skin vs. infected eczema (*p* = 0.187), infected eczema vs. crusted scabies on scalp (*p* = 0.056). Statistically significant differences (*p* < 0.05) were observed for every other pairwise comparison of proportions (Figure 3). Participants demonstrated a strong ability to correctly identify images that did not depict scabies (impetigo/boil, 84% correct, dry skin, 93% correct). The exception was an example of eczema, which only 55% correctly identified as a non-scabies case. The identification of images that depicted ordinary scabies was variable depending on clinical presentation. Participants could identify the ‘classical’ presentation of scabies (papular lesions, burrows visible on the wrist) with high confidence (90% correct). Other presentations of ordinary were identified with less confidence, including dry excoriation with generalized rash in the axillary region (68% correct) and ordinary scabies on elderly skin (55% correct). The ability of respondents to correctly identify images depicting crusted scabies was low. Crusted scabies of the toes with superimposed bacterial infection was correctly identified by only 17.2%. Although 75.8% of respondents correctly believed that scabies could affect the scalp or the face in the knowledge question, only 31.6% of participants correctly identified the crusted scabies infection of the scalp in the image. Using McNemar’s test, the difference between these proportions was determined to be statistically significant (*n* = 96, *p* < 0.01). This indicates a discordance between theoretical knowledge of crusted scabies and practical ability in scabies management.

### Attitudes and perceptions towards scabies

Most respondents agreed that scabies outbreaks were a problem in aged care facilities (*W* = 2075.5, *n* = 110, *p* = 0.003), that scabies outbreaks caused disruption to facilities (*W* = 3,271.0, *n* = 108, *p* < 0.001), and felt uncomfortable at the prospect of catching scabies (*W* = 3,376.0, *n* = 110, *p* < 0.001) (Table 3). Although most participants expressed discomfort at the idea of catching scabies (median 4.00), they felt neutral interacting with scabies-infected residents (median 3.00) (Table 3). The distribution of all rating scale responses demonstrated that participants who had been exposed to ordinary scabies, crusted scabies, or scabies outbreaks had no significant difference in attitudes to those that had not (*p* > 0.05). We asked respondents which resources they would find useful in learning more about scabies and its control. In-person workshops and training were the most selected resource (*n* = 73, 57%), followed by online resources (*n* = 69, 53.9%) and the provision of formal written guidelines or standard operating protocols (*n* = 67, 52.3%). Online training modules were the least popular resource option (*n* = 31, 27.9%).

**Table 3.** Likert-style responses on perceptions about scabies

Question	Median score (1–5)	n
How much of a problem is a scabies outbreak in an aged care facility	3.00	110
Scabies outbreaks cause disruption to facility operations	4.00	108
Personally, how do you feel about dealing with residents with scabies	3.00	110
How would you feel if you caught scabies (or, if you have had scabies, how did you feel?)	4.00	110

## Discussion

Worldwide, scabies prevention and treatment within aged care facilities is inadequate, and outbreaks remain a leading cause of morbidity within these settings [9]. When strategizing interventions for the improvement of scabies management, it is important to explore the driving factors of outbreaks. It has been previously demonstrated that misdiagnosis or delayed diagnosis of index cases of scabies underlies most outbreaks [6]. Under resourcing of workers and workload constraints may also be a driving factor in scabies outbreaks. The relative influence of each exacerbating factor in this multifaceted issue remains understudied. This study examined 128 mail-in surveys completed by aged care facility workers, examining the experience, knowledge, and attitudes of workers in relation to scabies prevention and treatment.

To investigate the theoretical knowledge of workers, a construct score was developed using 11 knowledge-based multiple-choice questions from the survey. While construct scores demonstrated a reasonably high level of knowledge, score distributions were not significantly different between experience – both in regard to years worked in the sector and previous exposure to scabies patients and/or outbreaks. This was somewhat unexpected and could suggest a lack of learning within the facilities, with new residential care workers demonstrating similar levels of knowledge compared to more experienced workers. Construct score distributions were however affected by participant age, implying more experience and knowledge on scabies occurring throughout life. Other demographic details, such as the role within the facility and education status, did not influence knowledge construct scores. It could be speculated that overall changes in community scabies exposure and declining general awareness/knowledge in recent years could explain why knowledge was associated with age but not specific aged-care experience.

To our knowledge, this represents the first survey of scabies knowledge, attitudes, and experiences of practitioners in aged-care facilities, despite the relatively common occurrence and substantial health burden imposed by scabies in this setting. Compared to practitioner surveys in other settings internationally, our participants displayed similar or higher levels of general scabies knowledge, although this may have been inflated by limitations of our survey design. A survey of physicians in Saudi Arabia (*n* = 216) showed inadequate levels of knowledge regarding scabies diagnosis and management, with an overall median knowledge score of 67.5% [10]. Similarly, a Belgian study identified knowledge scores of 59% amongst general practitioners, with dermatologists demonstrating higher scores, as expected (79%) [11]. A mixed-methods survey in Guinea-Bissau, consisting of community members and healthcare workers, showed high levels of general awareness but poor diagnostic capability (42% positive identification of scabies lesions) and misconceptions around scabies aetiology and transmission [12].

Although our respondents demonstrated a good theoretical knowledge of scabies, their ability to identify scabies cases in a practical scenario was inconsistent. When provided with eight images of dermatological skin conditions, participants were asked to identify which images represented scabies infections. The proportion of correct answers ranged from 17% (crusted scabies) to 93% (non-scabies, dry skin). Participants demonstrated a higher level of specificity than they did sensitivity – non-scabies cases were generally associated with a higher proportion of correct identification than scabies cases. The non-scabies presentation with the lowest diagnostic confidence (55%) depicted an eczema flare. This is notable because eczema (atopic dermatitis) is a frequent differential diagnosis for crusted scabies, and the initial incorrect application of corticosteroids is a key contributor to the development and exacerbation of crusted scabies [6, 13]. These survey results highlight the difficulty in differentiating atopic dermatitis and scabies and should be an area of focus for future education. One caveat to our interpretation of diagnostic specificity is that the survey design meant that a designation of ‘non-scabies’ by the respondent was presumed (by the absence of tick or circle) unless otherwise specified. This meant that uncertain respondents may have been more likely to identify a condition as ‘non-scabies’ than ‘scabies’. There was also a lower response rate for this component of the survey, with only 99/128 participants completing this element of the survey – this could indicate a lack of confidence or clarity with the task. Future survey design should have more explicit selection options.

The image depicting pathognomonic signs of scabies on the wrist was associated with a high proportion of correct answers (90%), confirming that respondents had familiarity with scabies within specific contexts. However, other images that also showed ordinary scabies infections had lower proportions of correct answers (55% and 68%). Interestingly, one image showed ordinary scabies affecting elderly skin with numerous scratch marks, which could be a common clinical manifestation in an aged care facility, noting that elderly patients may present with atypical signs of scabies [3]. However, this case was largely misidentified. Previous literature has noted that such signs of scabies may be seen as attributable to generalized dry skin or ‘senile itching’, which is not uncommon in this setting [14]. In addition to the reduced capacity of residents to voice discomfort [5], it is likely that cases such as these facilitate scabies outbreaks. As well as allowing transmission, missed scabies diagnoses are associated with poor clinical outcomes for the individual, especially if crusted scabies develops from iatrogenic corticosteroid use.

Crusted scabies cases were associated with the lowest proportions of correct identification by respondents. The image depicting infected crusted scabies of the feet and toenails had only 17% correct identification, a concerning finding given that infections from crusted scabies can become life-threatening [15]. The second image of crusted scabies impacting the scalp was also not readily identified (32% correct), with differential diagnoses commonly including psoriasis or seborrheic dermatitis [16]. A persisting misconception of scabies is that areas of the scalp and face are rarely affected, which was supported by 33% of our respondents. Indeed, some current guidelines for topical treatment of scabies only recommend treating the skin from the neck down [17, 18]. While this may be the case for most presentations of ordinary scabies, crusted scabies in atypical locations commonly present in the elderly and immunocompromised index cases [19].

Delayed diagnosis is a common feature of scabies outbreaks in residential facilities. In a UK study, Phipps and colleagues [7] reported a median time of 6 weeks between initial symptom onset and outbreak notification. During this time, scabies outbreaks continue to spread, assisted by the characteristic delay in the appearance of symptoms post-exposure in a primary infestation of scabies (usually 4–6 weeks) [2]. The lower proportion of correct responses to this survey question suggests limited awareness of this delayed onset, which may contribute to spread and impact decision-making about who receives treatment when scabies is identified [9]. A similar lack of knowledge regarding incubation periods has been reported in other practitioner surveys [10].

There was also uncertainty amongst our respondents about appropriate diagnostic methods. While most participants correctly understood that a negative skin scraping does not preclude a diagnosis of scabies, they still believed that skin scrapings were likely to be the most sensitive method for detecting scabies. In practice, the diagnostic sensitivity of skin scrapings and microscopy is low (<50%) [3], and clinical presentation and history is currently considered the most sensitive method for diagnosis despite its lack of specificity [20]. The development of more sensitive, specific, and non-invasive diagnostic methods is a priority area for scabies control [21], and debate exists around diagnostic methods in residential care settings [22, 23].

Scabies, and especially crusted scabies, is an often-stigmatized condition. This was reflected in our survey, with 40% of respondents believing that the statement ‘poor hygiene causes people to develop scabies’ was true. This was further demonstrated by responses to the Likert-style question ‘How would you feel if you caught scabies?’ Respondents had a median response of 4 on a scale of 1 to 5, which demonstrated that they felt uncomfortable with the idea of contracting scabies. The interpretation here could be limited to the way respondents interpreted the question (e.g., physical discomfort vs. emotional distress/stigma). This potential stigma aligns with findings reported from other studies [7, 12, 24] and likely contributes to underreporting at both the individual and facility level, a consideration when planning interventions.

Although rich insights have been obtained through this work, our survey design was not without its limitations. Participants were not supervised during the completion of the survey, meaning that some of the responses may not represent individuals but rather have been discussed amongst participants. This could have led to an overinflation of knowledge construct scores and proportion of correct responses to individual questions. Similarly, the voluntary nature of the survey may have led to an over exaggeration of scabies knowledge, as workers with higher levels of knowledge may have been more likely to undertake the survey. Conversely, the above noted stigma surrounding scabies may have led to underreporting in particular questions, despite the anonymity of the survey. As with many cross-sectional surveys, questions were routinely skipped by respondents, and some surveys were returned with missing pages, especially for the final section on clinical identification. The number of missed values limits our interpretation of some results. Specifically, knowledge construct scores may be overinflated as they were selected for participants giving complete responses, thus likely having higher confidence and knowledge about scabies. However, similar trends were shown when non-respondents were included in the calculated proportions of individual questions. These issues could have partially been alleviated by using an electronic survey design, but the paper format was selected for increased visibility, engagement, and ease of completion.

## Conclusions

Although there was a suitable level of theoretical knowledge amongst aged care workers, particularly around ordinary scabies, workers demonstrated minimal ability to identify atypical presentations of scabies and particularly crusted scabies. This limited knowledge is likely a key factor in the development of outbreaks from misdiagnosed or undiagnosed index cases of crusted scabies. The dissemination of these results to facilities will now form the basis for the co-design of educational resources. This highlights potential for before and after surveys to test effectiveness for such resources. A comprehensive control program that focusses on staff education about the diagnosis and treatment of scabies may significantly lower the incidence of disease and help prevent future outbreaks. Control guidelines should be relevant for managing scabies in elderly patients and readily accessible to target workers.

## Supporting information

Lightbody et al. supplementary material Lightbody et al. supplementary material

## Data Availability

All relevant data are presented in the manuscript and supplementary material. Images used in the survey are not published due to copyright restrictions but can be disseminated for educational purposes upon request to the corresponding author.

## Supplementary material

For supplementary material accompanying this paper visit http://doi.org/10.1017/S0950268824001377. click here to view supplementary material
