# Supplementary material for: A cross-sectional survey of knowledge and attitudes towards scabies control in Australian aged care facilities
Source: Epidemiol Infect. 2024 Oct 21;152:e129. doi: 10.1017/S0950268824001377 (PMC11502422; doi:10.1017/S0950268824001377)
Supplement: Lightbody et al. supplementary material [file S0950268824001377sup001.docx]

Instructions:

Thank you for taking the time to participate in this survey. We hope that the information provided will help us understand more about the occurrence of scabies in aged care settings, and assist in the development of new educational resources which will benefit staff and residents alike.

We estimate the survey will take 10-15 minutes to complete. Your responses are anonymous, and cannot be linked back to your facility. To get the most accurate information possible, we ask that you answer questions individually, honestly and to the best of your knowledge. Responses can be placed in the reply-paid envelopes provided and either mailed in, or placed in the secure collection box provided.

*Your contribution to this research is much appreciated.*

Part 1: About you, and your experiences with scabies

| **1. What is your main role in aged care?** | 🞏 Patient care assistant_1_  🞏 Enrolled/Registered Nurse_2_  🞏 Allied Health practitioner_3_  🞏 Support services_4_  🞏 Management/Administration_5_  🞏 Physician_6_  🞏 Other (please specify)_7_: | |
| --- | --- | --- |
|  |  | |
| **2. How long have you worked in aged care?** | 🞏 Less than 1 year_1_  🞏 1-5 years_2_  🞏 5-10 years_3_  🞏 10-20 years_4_  🞏 20+ years_5_ | |
|  |  | |
| **3. Do you work with high care patients requiring physical assistance?** | 🞏 Never_1_  🞏 Rarely_2_  🞏 Sometimes_3_  🞏 Often_4_  🞏 Always_5_ | |
|  |  | |
| **4. Your education** | 🞏 Did not complete high school_1_  🞏 Completed high school_2_  🞏 Diploma/Certificate qualification_3_  🞏 Bachelors university qualification_4_  🞏 Post-graduate qualification_5_ | |
|  |  | |
|  |  | |
| **5. Your age group and gender** | 🞏 18-25_1_ |  |
|  | 🞏 25-40_2_ | 🞏M_1_ / F 🞏_2_ |
|  | 🞏 40+_3_ |  |
|  |  | |
| **6. While working in aged care, have you encountered?**  (tick all that apply) | 🞏 Individuals with ordinary scabies?_1_  🞏 Individuals with severe scabies (also known as Norwegian or crusted scabies)?_2_  🞏 Several people or staff within a single facility with scabies (scabies outbreak)_3_ | |
| **If yes, how many times?** | 🞏 Once_1_ 🞏1-5 times_2_ 🞏 more than 5 times_3_ | |
| **How recently?** | 🞏 Last 6 months_1_ 🞏 Last 2 years_2_ 🞏 Last 10 years_3_ | |
| **7. Have you ever caught scabies through your work?** | 🞏 Yes_1_ / No_2_ 🞏 | |
| **Do you have colleagues that have caught scabies through their work**? | 🞏 Yes_1_ / No_2_ 🞏 | |
| **Have members of your family caught scabies through your work?** | 🞏 Yes / No 🞏 | |
|  |  | |

**Part 2: Scabies Transmission, control and diagnosis**

Please complete the following multiple-choice questions

| 1 | Scabies is most commonly transmitted by | |
| --- | --- | --- |
|  | a. | Skin to skin contact |
|  | b. | Animals |
|  | c. | Furniture |
|  | d. | Clothing |
|  |  |  |
| 2 | Poor hygiene causes people to develop scabies | |
|  | a. | True |
|  | b. | False |
|  |  |  |
| 3 | The following factors are important to prevent scabies transmission | |
|  | a | Isolating the patient |
|  | b | Barrier nursing |
|  | c | Treatment of all people in contact with the patient |
|  | d | All of the above |
|  |  |  |
| 4 | Bedding from a scabies patient is best treated by | |
|  | a. | Insecticide treatment |
|  | b. | Incineration |
|  | c. | Hot water washing and drying |
|  |  | |
| 5 | The following are effective treatments for scabies | |
|  | a. | Scabicidal cream applied to the entire body |
|  | b. | Scabicidal cream applied to affected areas |
|  | c. | Anti-inflammatory / steroid creams |
|  | d. | Alcohol based hand sanitizer gels |
|  |  |  |
| 6 | Itching is the most common symptom of scabies | |
|  | a. | True |
|  | b. | False |
|  |  |  |
| 7 | If mites are not found in skin scrapings, the patient does not have scabies | |
|  | a. | True |
|  | b. | False |
|  |  | |
| 8 | Scabies is most likely to be detected by | |
|  | a. | Patient symptoms and history |
|  | b. | Skin scrapings |
|  | c. | Blood test |
|  | d. | Detection of mites in bedding and clothing |
|  |  |  |
| 9 | **Scabies never affects the scalp or face** | |
|  | a. | True |
|  | b. | False |
|  |  |  |
| 10 | **Clinically, scabies presents in the following way/s** | |
|  | a. | A characteristic ‘burrow’ line on the skin |
|  | b. | Pimple like rash |
|  | c. | Dry skin |
|  | d. | Scaly or scabby skin |
|  | e. | All except c. |
|  |  |  |
|  |  | |
| 11 | **Scabies symptoms appear quickly after contact with an infected person** | |
|  | a. | True |
|  | b. | False |
|  |  |  |

|  |  |  |
| --- | --- | --- |
| **Part 3: Your feelings about scabies** | | |
|  |  | |
| 14 | **On a scale of 1-5, overall how much of a problem is a scabies outbreak in an aged care facility? (1=not a problem at all, 5= extremely big problem)** | |
|  | 1 2 3 4 5 | |
|  |  | |
| 15 | **Scabies outbreaks cause disruption to facility operations (1=none at all, 5=serious disruption)** | |
|  | 1 2 3 4 5 | |
|  |  | |
| 16 | **Personally, how do you feel about dealing with residents with scabies (1= not bothered, 5= extremely uncomfortable)** | |
|  | 1 2 3 4 5 | |
|  |  | |
| 17 | **How would you feel if you caught scabies (or, if you have had scabies, how did you feel)? (1=not bothered, 5= extremely uncomfortable** | |
|  | 1 2 3 4 5 | |
|  |  | |
| 18 | **What resources would you find useful for obtaining information about scabies and its control?**  **Tick all those that apply** | |
|  | 🞏 Workshops / training (i.e face-to-face)_1_ 🞏 tablet / smart phone application /e-training course_4_ | |
|  | 🞏 Posters / flipcharts available in facility_2_ 🞏 formal written guidelines / operating protocols_5_ | |
|  | 🞏 The internet / online resources_3_ | |
|  |  | |
| 19 | **Any other comments?** | |
|  |  | |
|  |  | |
|  |  | |
|  |  | |
|  |  | |

**Part 4: Is it scabies?**  Diagnosis of scabies is tricky, even for experienced dermatologists. Which of these pictures show scabies? Tick all that apply. Good luck!

Descriptions only provided- these images used in the survey can not be published due to copyright restrictions but can be shown on request.

Image A: Two boils on forearm, staphylococcal impetigo, otherwise clear skin

Image B: Ordinary scabies- elderly skin showing scratch marks

Image C: Dry/peeling skin- sunburn

Image D: Crusted scabies- yellow plaques on toes with superimposed bacterial infection

Image E: Ordinary scabies: dry excoriated and mild hyperkeratosis of axilla region, with generalized rash on torso

Image F: Eczema, some serous yellow crusting indicating secondary infection

Image G: Crusted scabies, yellow/whitish plaques on scalp

Image H: Ordinary scabies, papular lesions and burrow marks on wrist
